# Fabrication of stainless-steel microfibers with amorphous-nanosized microstructure with enhanced mechanical properties

**DOI:** 10.1038/s41598-022-14475-5

**Published:** 2022-06-24

**Authors:** Elham Sharifikolouei, Baran Sarac, Yonghui Zheng, Piotr Bala, Jürgen Eckert

**Affiliations:** 1 Department of Applied Science and Technology, Politecnico di Torino (POLITO), Corso duca Degli Abruzzi 24, 10129 Turin, Italy; 2 Erich Schmid Institute of Materials Science, Austrian Academy of Sciences, Jahnstraße 12, 8700 Leoben, Austria; 3 Department of Electronics, Key Laboratory of Polar Materials and Devices (MOE), East China Normal University, Shanghai, 200241 China; 4 AGH University of Science and Technology, Faculty of Metals Engineering and Industrial Computer Science, 30 Mickiewicza Ave, 30-059 Krakow, Poland; 5 Academic Center for Materials and Nanotechnology, AGH University of Science and Technology, 30 Mickiewicza Ave, 30-059 Krakow, Poland; 6 Chair of Materials Physics, Department of Materials Science, Montanuniversität Leoben, Jahnstraße 12, 8700 Leoben, Austria

**Keywords:** Synthesis and processing, Metals and alloys

## Abstract

Metallic glasses (MG) have attracted much attention due to their superior hardness and good corrosion resistance. However, designing new MG compositions is still a big challenge, and their integration into different systems is limited when they are in the shape of bulk materials. Here, we present a new method for the fabrication of MG in the form of microfibers which could greatly help them to be integrated within different systems. The newly proposed technique has the ability to form MG structure from commercially available alloy compositions thanks to its significantly improved quenching rate(~ 10^8^ K.s^−1^). In this technique, individual melt droplets are ejected on a rotating wheel forming a thin film which are ruptured upon solidification leading to the formation of MG microfibers. In this regard, we have fabricated microfibers from a commercial DIN 1.4401 stainless-steel which could form a completely amorphous structure confirmed by DSC, XRD, and HRTEM. The fabricated MG microfibers show an increased hardness for more than two-fold from 3.5 ± 0.17 GPa for the as-received stainless-steel to 7.77 ± 0.60 GPa for the amorphous microfibers. Subsequent heat-treatment of the microfibers resulted in a nanocrystalline structure with the presence of amorphous regions when the hardness increases even further to 13.5 ± 2.0 GPa. We propose that confinement of both shear transformation zones and dislocations in the heat-treated MG microfibers plays a major role in enhancing strength.

## Introduction

The invention of melt spinning to rapidly solidify liquid metal jets on a rotating copper wheel (at cooling rates of 10^4^–10^7^ K.s^–1^) and form ribbons with a thickness of 10–50 µm has driven the discovery and development of metallic glasses over recent decades^1^. Because they exhibit improved properties compared with their crystalline counterparts, including hardness and strength, corrosion and wear resistance and excellent soft magnetic properties, metallic glasses have remained the focus of considerable interest in fundamental and applied research^2–4^. Most alloy systems used to generate metallic glasses by melt spinning are binary or ternary alloys with deep eutectic points. In principle, the eutectic composition generally has a low melting point and, therefore, a stable liquid phase. This is in line with Bernal’s dense random packing model for metallic glasses, which considers them to be frozen metallic liquids^5^. (Conventional) melt spinning generates only ribbons or sheets, thus limiting the application of metallic glasses to specific geometries. This has triggered the search for compositions allowing fabrication of metallic glasses in bulk form and focusing on alloys with deep eutectic points. However, since the eutectic transitions are largely unknown for multicomponent alloys (more than four elements), the identification of new glass-forming compositions relies mostly on trial and error, and finding new glass-forming compositions is still a major challenge^6^.

Metallic glasses in the form of microfibers offer excellent structural, and functional properties due to their unique microstructure and size effects^7,8^. Both theoretical and experimental studies indicate that plasticity of metallic glasses can be improved if their thickness decreases^9,10^. In this regard, the toughness of micro-and nanoscale metallic glass fibers is higher than their bulk form. Additionally, they can be used in many applications because they can be easily formed or woven into cellular structures, bundles, textiles, and smart sensors^11,12^. In fact, there are plenty of work on the fabrication of metallic glass nanofibers but limited research is dedicated to the fabrication of metallic glass microfibers that could be directly utilized or embedded in other applications. For the fabrication of metallic glass nanofibers, there are top-down and bottom-up approaches^13^. In bottom-up approaches, both physical and chemical synthesis are employed. Nakayama et al. has shown a physical method for fabrication of metallic glass nanowires with the diameters in the 50–2000 nm range via gas atomization^14^. In the chemical synthesis rout, the process is based on the chemical reduction methods. The advantage of this method is that it does not require fast cooling techniques and can be achieved even when the glass forming ability of an alloy is low. The size of the metallic glass nanostructures by the chemical reduction method can range from 2 nm to several hundred nanometers^15,16^. Electrochemical modification of metallic glasses is a populare top–down approach to create nanostructured membranes. Lie et al. has reported the fabrication of Cu–Ag bimetallic porous nanomembrane through dealloying of multicomponent metallic glass^17^. Moving toward the fabrication of metallic glass microfibers, one of the most common ways is via force driving method where metallic glass master alloy (rod) is rapidly heated into its supercooled liquid region, and the pre-applied force leads to microscale metallic glass microfibers via superplastically deformation of the metallic glass^8^. In a more recent attempt in the creation of metallic glass microfibers, Liu et al. has fabricated series of Gd-Al-Co-Fe based metallic glass microfibers with diameters in the range of 30–45 µm and a length of more than 500 mm, using a precision rotated-dipping device^7^. In this work, we have used a modified melt spinning technique for the very first time to fabricate metallic glass microfibers from a conventional multicomponent alloy, DIN 1.4401 stainless steel. The higher quenching rate in the modified technique allows fabrication of metallic glass microfibers from alloys with low glass forming ability.The resulting metallic glass microfibers are in the range of 2–20 µm in diameter with the length from several mm to 1000 mm.

Among the most notable properties of metallic glasses are their extremely high strength and hardness, which make them perfect candidates for applications in which strength is of crucial importance. The deformation characteristics and mechanisms of crystalline metals and alloys are well understood and governed by formation, movement and interactions of dislocations. However, for metallic glasses, in which there is no long-range atomic order, the deformation mechanism is completely different. The mechanical properties of metallic glasses depend on their chemical composition, and this suggests a strong correlation between atomic (and electronic) arrangements in the alloy^6,18^. The lack of long-range order in metallic glasses means that any change in the vicinity of the atom cannot take place by low-energy processes such as dislocation movements, and any rearrangement in the local area requires a relatively high amount of energy and stress. The bonding characteristics in metallic glasses are among the key factors used to resolve some fundamental issues in metallic glasses (MGs), such as deformation, relaxation and the glass transition^19–22^. Unlike conventional crystalline metals, the macroscopic elastic modulus in a metallic glass depends on the atomic bonding strength and on the atomic configuration and atomic packing density, including short- and medium-range order^23–25^. Plastic deformation in metallic glasses is believed to correlate with the formation of local atom clusters in what are referred to as shear transformation zones “STZ”^26–28^. The transition from local shearing to macroscopic shear bands arises when the generated mechanical energy increases atomic mobility dramatically. If the internal energy accumulated from elastic or anelastic deformation reaches a critical value, it may lead to softening along shear planes^6^. Therefore, yielding and shear band formation in BMGs are intrinsic processes related to shear stress-induced glass-to-liquid transition.

One way to prevent or inhibit the propagation of shear bands is to introduce second phases (mostly crystalline) through the fabrication of dual-phase composites. Additional benefits are realized when implementing this approach. The second phase can reduce strain localization and thus improve ductility, where brittle fracture is one of the major limitations of BMGs^29–32^. In addition, the second phase enhances the toughness of BMGs^33,34^. The underlying mechanism for this involves modification of the microstructure to hinder the propagation of shear bands. Much research on the creation of such microstructures has focused on introducing precipitated particles as a second reinforcing phase. For example, Zhang et al.^34^ successfully implemented this approach in generating high-strength-high-fracture toughness Zr-based BMGs. One of the most promising results for dual-phase composites is the introduction of a crystalline dendritic phase into the amorphous matrix via in situ precipitation during melt solidification^35,36^.

Here, we report metallic glass microfibers made of commercial grade DIN 1.4401 stainless steel via a customized melt spinning technique. Formation of an amorphous structure was confirmed by X-ray diffraction (XRD), differential scanning calorimetry (DSC), and high resolution (scanning) transmission electron microscopy (HR(S)TEM). Subsequent heat treatment of the microfibers resulted in the generation of nanocrystalline grains protected by an amorphous layer on the surface. Moreover, we used nanoindentation to probe the hardness of the as-cast amorphous stainless steel and the hardness of heat-treated stainless steel with a nanocrystalline/amorphous structure and propose a relationship between the microstructure and mechanical properties of this composite material.

## Results

### Fabrication of stainless steel microfibers

**Figure 1:**
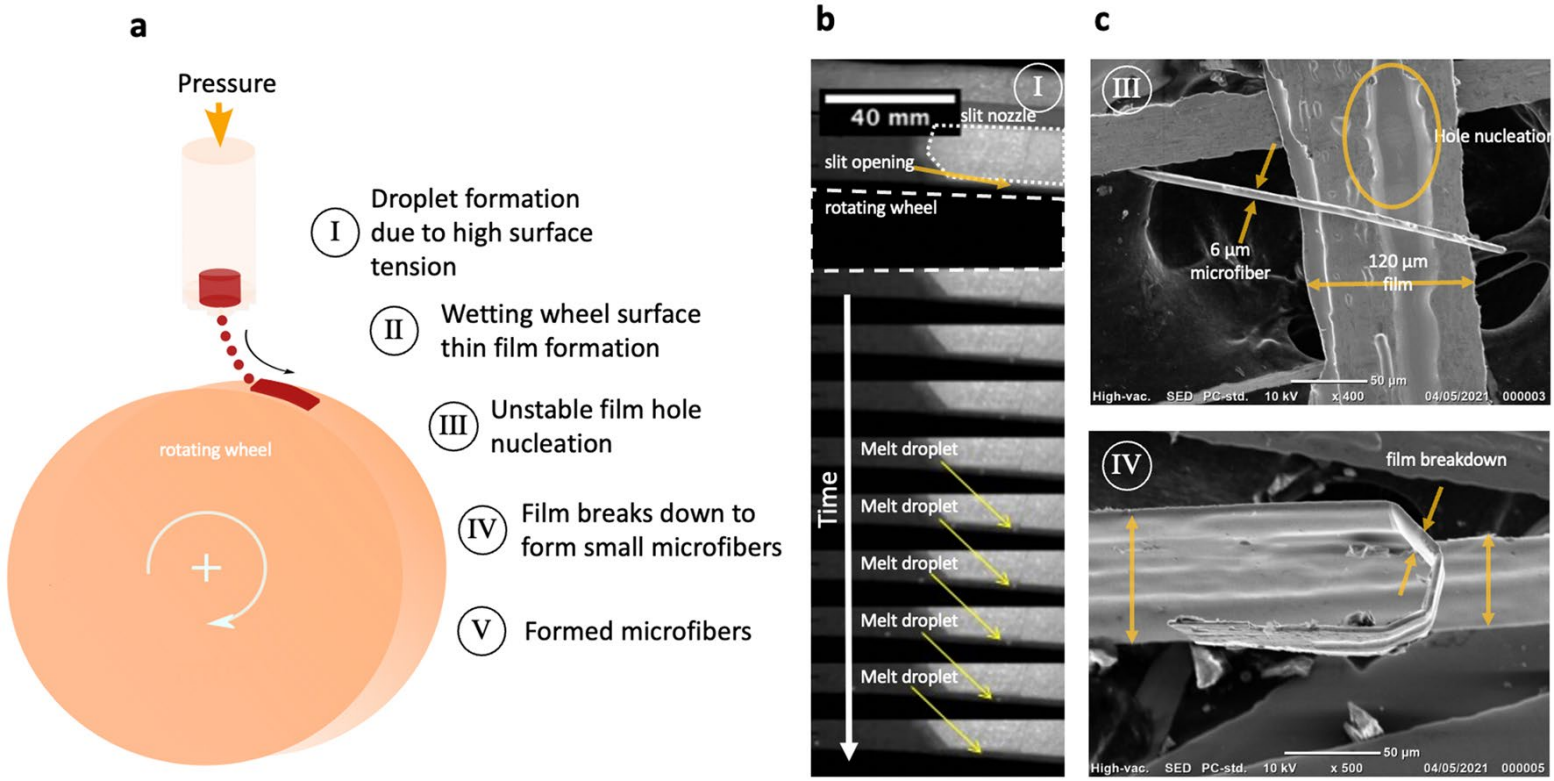
Fabrication of stainless steel microfibers by modified melt-spinning technique. (**a**) Schematic representation of metal microfiber formation by the modified melt-spinning technique: (I) Droplet formation due to high surface tension (II) Wetting wheel surface thin film formation (III) Unstable film hole nucleation (IV) Film breaks down to form small microfibers (V) Formed microfibers (**b**) Images taken from a high-speed camera movie during the melt-spinning process (republished from Sharifikolouei dissertation) (**c**) SEM images of the unbroken stainless steel film vs. stainless steel microfibers fabricated by melt-spinning.

Figure 1a provides a schematic illustration of the process used for microfiber formation by the modified melt-spinning device. In contrast to stationary puddle formation between the slit nozzle and the rotation wheel in the classic planar flow melt spinning technique, the new method avoids puddle formation. The slit nozzle opening, which is considerably smaller than the slit nozzle opening for ribbon formation, is limited to 30 µm. Due to the very high surface tension of molten stainless steel at 1823 K, melt droplets are formed on the slit nozzle opening. Li et al.^37^ have previously measured surface tensions between 1500 and 1800 mN.m^−1^ for several ferritic stainless steel melts within this temperature range. Each droplet is ejected separately by applying Ar gas pressure on the melt. Due to the fast nature of melt ejection, the casting process was observed, and images were captured by a high-speed camera during the experiments (see Fig. 1b). Each droplet wets the rotating copper wheel, and wheel rotation helps the melt create a thin film on the wheel surface. The film spontaneously breaks down into smaller parts and simultaneously solidifies in the microfiber form. The exact mechanism of microfiber formation is not clear. However, the analysis of some defective stainless steel products shown in Fig. 1c reveals that the unbroken film looks more like a microribbon, suggesting that the film breaks down into smaller regions due to hole nucleation and growth. The formation of a hole in the thin film depends on a number of parameters, including the wheel surface roughness, the vibrational forces applied on the film as a result of the high wheel frequency, and centripetal forces.

**Figure 2:**
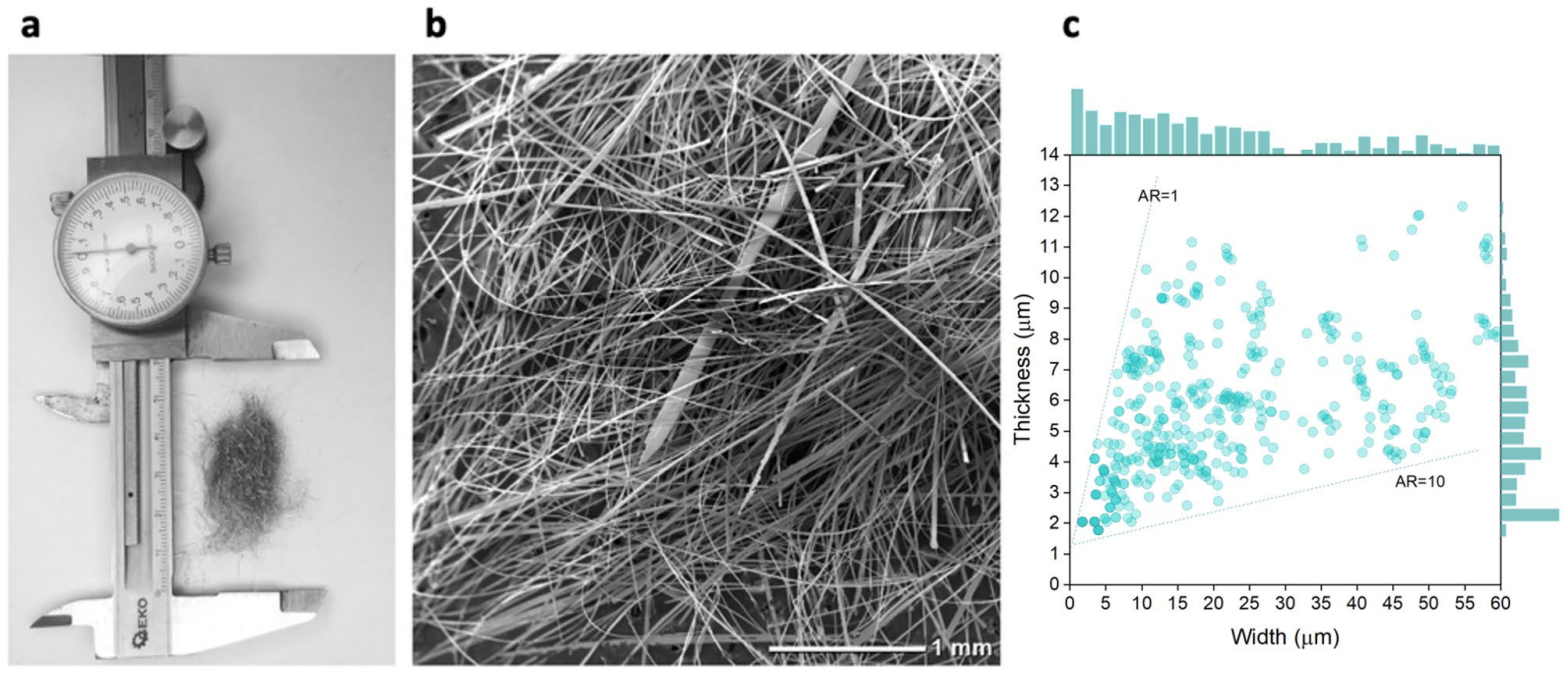
Microfibers fabricated by the modified melt-spinning technique. (**a**) Appearance of metal microfibers fabricated by the modified melt-spinning technique. (**b**) SEM image of the fabricated metal microfibers. (**c**) Size distribution of the microfibers. The microfibers have rectangular cross-sections, and their size distribution is characterized by their width and thickness. On top, the frequency histogram of the width distribution is presented; on the right side, the frequency histogram of the thickness distribution is presented.

Figure 2a shows the appearance of microfibers and Fig. 2b shows an SEM image obtained at higher magnification. The SEM images reveal that the produced microfibers exhibited a rectangular cross-section, and therefore, their size distribution was characterized by their width and thickness. Each data point in Fig. 2c shows the exact thickness and width of the microfiber cross-sections. The width-to-thickness ratio noted as the “aspect ratio” (AR) varied between 1 for perfectly round microfibers and 10 for more flat-shaped microfibers. On the top x-axis, the size distribution of the width is presented with the corresponding frequency count histogram (bin size = 2). On the right side of the y-axis, the thickness distribution is presented by a frequency count histogram (bin size = 0.5). The mean thickness value was 5.4 µm$\pm 2.3$.

### Characterization of stainless steel microfibers

**Figure 3:**
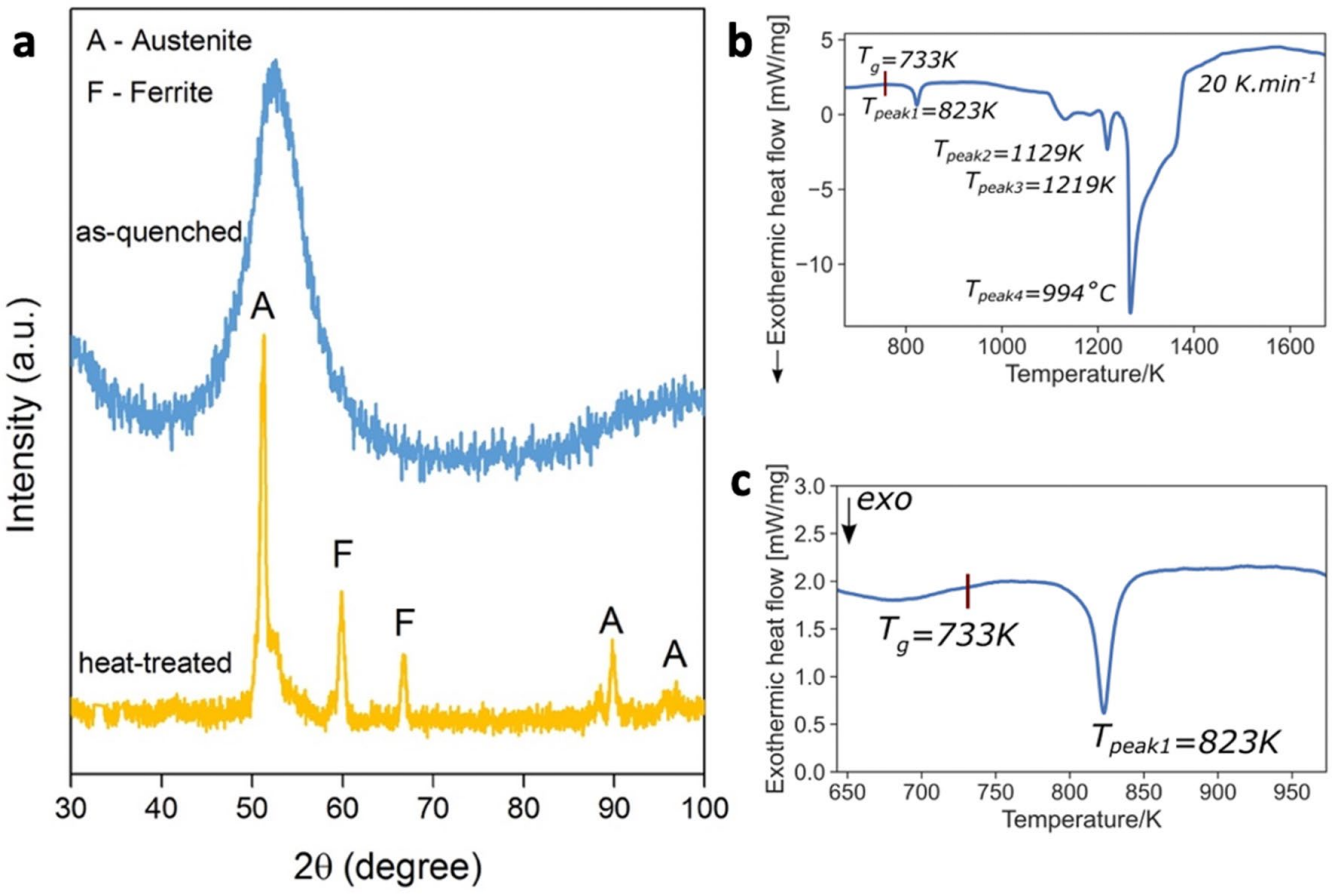
XRD and DSC analyses of as-quenched and subsequently heat-treated stainless steel microfibers. (**a**) XRD analysis of as-quenched 1.4401 stainless steel microfibers and subsequently heat-treated microfibers. (**b**) DSC data (heating rate 10 K.min^-1^) for the as-prepared DIN 1.4401 stainless steel microfibers. (**c**) DSC data magnified for the glass-transition region. The glass transition temperature is obtained at the inflation point.

Figure 3a shows an XRD analysis of the as-quenched DIN 1.4401 stainless steel microfibers after melt-spinning and a characteristic diffractogram of subsequently heat treated microfibers. The heat treatment of amorphous stainless steel microfibers used stepwise heating at a rate of 20 K.min^−1^ up to 773, 873, 973, and 1073 K, and an isothermal holding time of 1 h for each step was followed by furnace cooling under vacuum. The XRD pattern for the as-quenched microfibers showed a broad diffuse pattern characteristic of an amorphous structure. Grudeva and Kanev^38^ have previously shown that by adding refractory metals, such as Ti and W, to stainless steel, it is possible to form amorphous Fe + W and Fe + Ti thin films with thicknesses in the range of 50 to 5000 nm. However, a fully amorphous structure was not observed for the as-received commercial stainless steel composition without the presence of W or Ti. The XRD pattern for heat-treated stainless steel microfibers indicated a fully crystalline structure containing mostly austenite and ferrite as the major phases.

To understand the significance of forming an amorphous structure in stainless steel, one must consider the glass-forming ability (GFA). The glass-forming ability of a metallic alloy system is often evaluated by considering the width of the supercooled liquid region △*T*_x_ = *T*_x_ –*T*_g_, by the reduced glass transition temperature *T*_rg_ = *T*_g_/*T*_l_, or by the parameter *γ* = *T*_x_/(*T*_g_ + *T*_l_)^29,39–41^ where *T*_x_ is the crystallization temperature, and *T*_g_ and *T*_l_ are glass transition temperature and liquidus temperature respectively . Based on our DSC findings shown, we estimated both the reduced glass transition temperature *T*_rg_ and *γ* to evaluate the glass-forming ability of DIN 1.4401 stainless steel. Figure 3b shows the DSC analysis up to 1700 K. Figure 3c shows the region where glass transition temperature is found for the as-cast microfibers. From this Figure, *T*_g_ was found to be 733 ± 2 K at the inflection point of the heat step, and this was followed by the first crystallization peak with a maximum at 823 ± 2 K. Considering the number of elements present in the stainless steel composition, multiple crystallization peaks in the DSC heating curve were expected. Assuming a melting temperature of 1644 K^42^, *γ* ≈$0.34$, and *T*_rg_ ≈ $0.44$, indicating a very marginal GFA. The highest known *T*_rg_ values range from 0.66 to 0.69^43,44^. Therefore, it is rather surprising to obtain a fully amorphous structure from a stainless steel composition. According to Davies^45^, the quenching rate required to form a metallic glass with a GFA of 0.3 is approximately 10^8^ K.s^−1^. To calculate the theoretical quenching rate in our melt spinning approach, we used the following equation (equation (1)):

1 $$\frac{dT}{dt}= \frac{{(T}_{ejection}-{T}_{g})\kappa }{{X}^{2}{C}_{p}\rho }$$

where $\kappa$ is the thermal conductivity, *T*_ejection_ is the ejection temperature of the molten liquid, *t* is the time, *X* is the cooling film (fiber) thickness, $\rho$ is the melt liquid density, and *C*_p_ is the specific heat capacity of the alloy. Table 1 shows the values used to calculate the theoretical quenching rate. By inserting our values from Table 1 into Eq. (1), we obtained a quenching rate of *dT.dt*^−1^ = 1.4 × 10^8^ K.s^−1^. This value is compatible with our prediction for the quenching rate required to create an amorphous structure in DIN 1.4401 stainless steel. Therefore, the quenching rate of the modified melt spinning technique was theoretically two orders of magnitude higher than the standard melt spinning quenching rate^46^.

**Table 1:** DIN 1.4401 stainless steel properties taken from a data sheet published by The World Material^47^. The mean value for the thickness of the microfibers (X = 4 µm) was used for the calculations.

Parameters	$\rho$	${C}_{p}$	$X$	$\kappa$	${T}_{g}$	${T}_{\mathrm{ejection}}$
Units	[g.cm^−3^]	[J.kg^−1^. K^−1^]	[µm]	[W.m^−1^. K^−1^]	[K]	[K]
DIN 1.4401 stainless steel microfibers	8	500	5.4	15	733 ± 2	1823 ± 2

### TEM analysis of stainless steel microfibers

**Figure 4:**
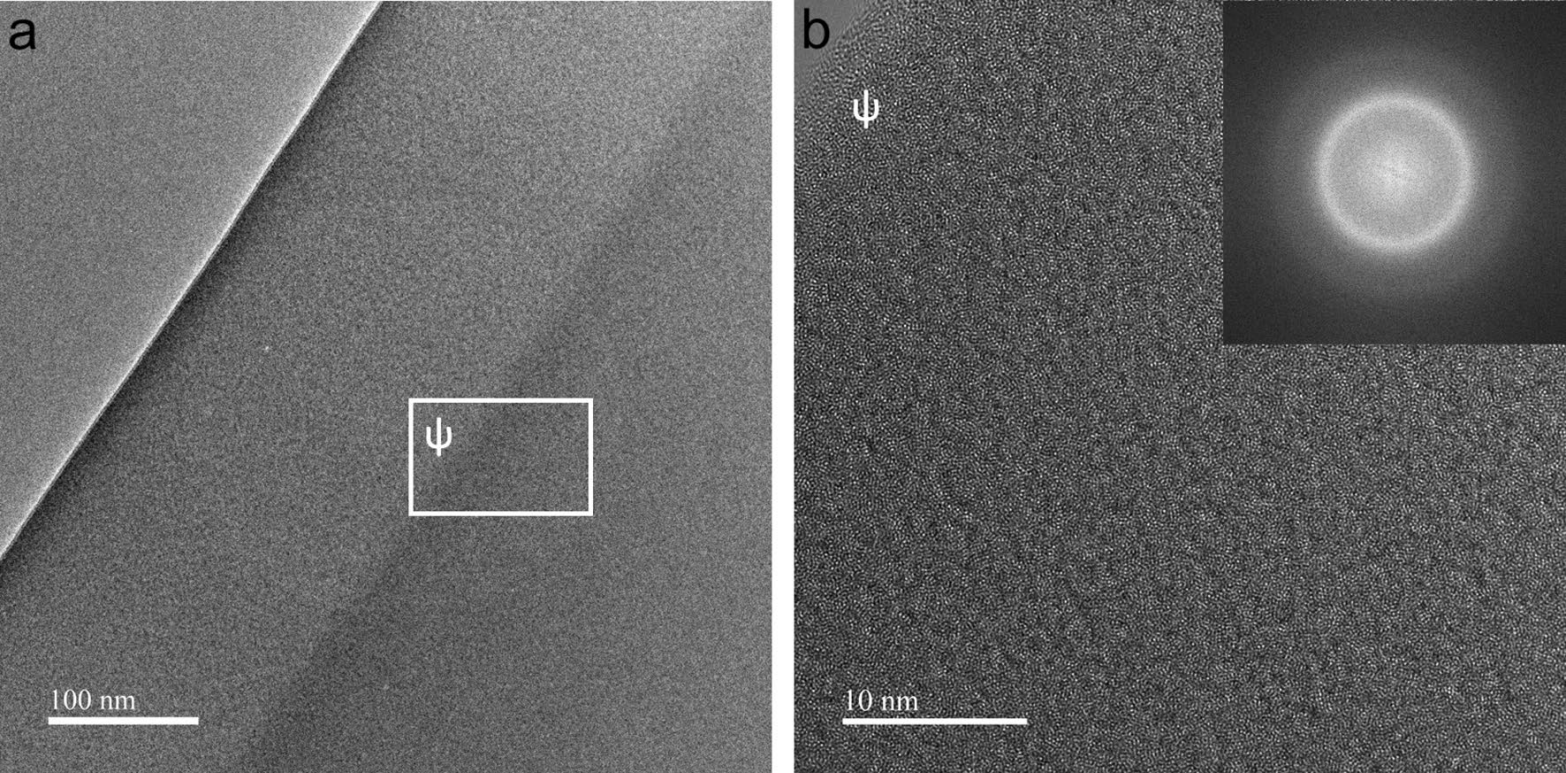
TEM analysis at the cross-section of the as-quenched stainless steel microfiber. (**a**) Bright-field image of an as-cast stainless steel microfiber cross-section. (**b**) The area marked by “ψ” is further magnified, and a fast Fourier transformation (FFT) confirms the amorphous structure (inset).

The as-quenched DIN 1.4401 stainless steel microfibers were further analyzed by transmission electron microscopy (TEM), as shown in Fig. 4. No crystalline grains were observed in the bright-field images of the as-quenched stainless-steel microfibers. The corresponding fast Fourier transformed (FFT) image in Fig. 4a shows a diffuse diffraction ring indicating a major amorphous phase in the fibers, which is in alignment with our previous XRD and DSC analyses. Because of the amorphous structure, the diffraction rings were quite broad due to disorder in atomic positions, and the width of the ring can reveal the short-range ordering of atoms^48^. A wider ring indicates more disorder in the arrangement of atoms. Elemental mapping was also conducted at the cross-section of the sample, which confirmed the homogeneity of the elemental distribution across the sample cross-section and is presented in Figure S1 in the Supplementary Information 1. The slight changes close to the surface were related to thinning during FIB preparation.

**Figure 5:**
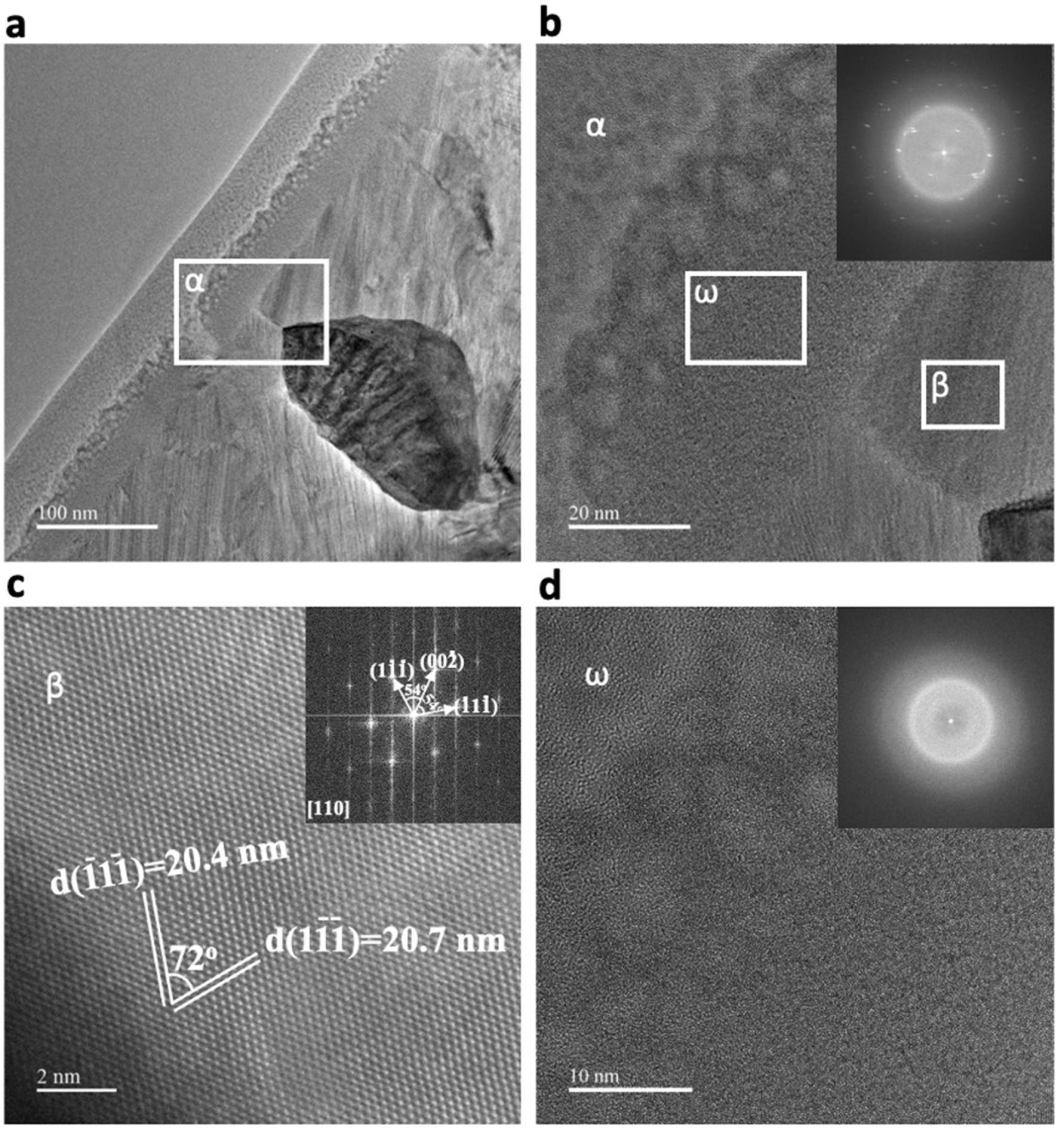
TEM analysis of stainless steel microfibers obtained by melt spinning after the subsequent heat treatment. (**a**) TEM bright-field image of the cross-section of a DIN 1.4401 stainless steel microfiber. (**b**) The area marked as “α”, close to the surface, was further magnified and investigated. The fast Fourier transformation of this area (“α”) shows the superposition of an amorphous ring and crystalline diffraction points (inset). (**c**) The TEM-BF image and its FFT on area “β” show a crystalline austenitic structure with an average spacing of 20.7 ± 2 nm. (**d**) The TEM-BF image and FFT from area “ω” (inset) indicate an amorphous structure.

The as-quenched amorphous stainless steel microfibers were heat-treated stepwise to 873, 973, and 1073 K with a heating rate of 20 K.min^−1^ and an isothermal holding time of 1 h for each step was followed by furnace cooling in vacuum. Figure 5a shows a BF TEM image from the cross-section of a FIB-prepared heat-treated stainless steel microfiber. The first result apparent after heat treatment is formation of nanometer-scale grains. The area marked as “α” was further magnified, and a fast Fourier transformation (FFT) image shows the superposition of austenite crystalline diffraction patterns and an amorphous diffusive ring (Fig. 5b and inset). Two different regions in area “α” were further investigated and are marked as “β” and “ω”. Area “β” shows a fully crystalline austenitic structure with an average spacing of 20.7 ± 2 nm (Fig. 5c). This is in line with our previous XRD analysis, in which austenite (Fe,Ni) was identified as one of the major crystalline phases. On the other hand, the area “ω” did not show a crystalline order, and the FFT image shows a diffuse pattern typical of an amorphous material.

**Figure 6:**
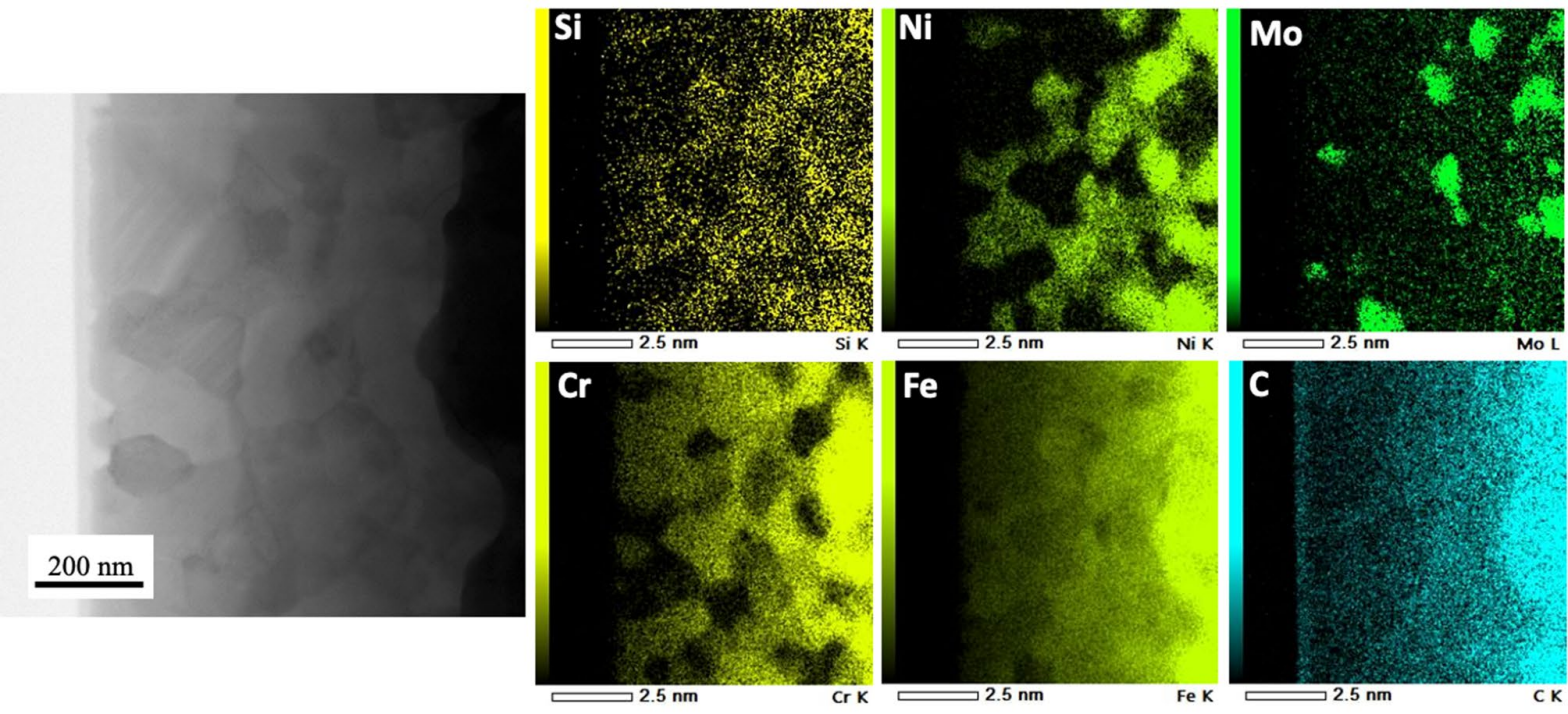
HAADF image of heat-treated stainless steel microfibers and the corresponding element maps.

Figure 6 shows elemental maps for the cross-section of heat-treated stainless steel microfibers. Using the linear intercept method, the average grain size was estimated to be approximately 80 nm based on the HAADF images. Furthermore, according to the map, some elements, such as Fe, Cr, and Ni, tended to accumulate in some grains, which is of no surprise. Ni tends to accumulate in austenitic grains. This could also explain why we observed a fully crystalline (austenite) structure in the region marked as “β” (Fig. 5), while the region “ω” (Fig. 5) had an amorphous structure.

### Nanoindentation on stainless steel microfibers

**Figure 7:**
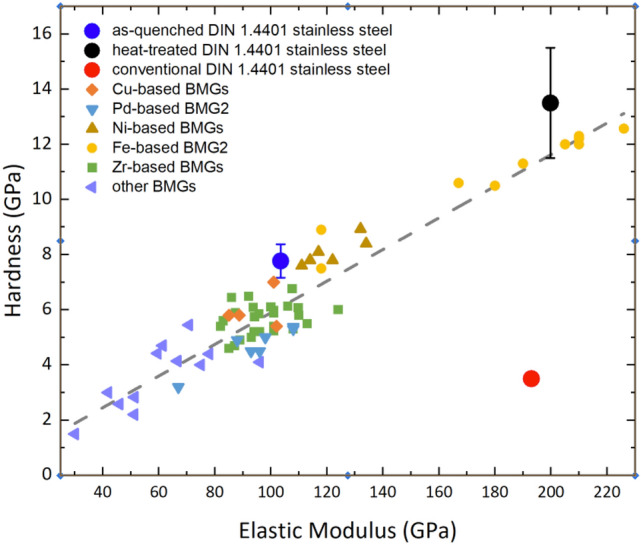
Hardness and elastic modulus of as-purchased DIN 1.4401 stainless steel, as-cast amorphous stainless steel microfibers, and heat-treated microfibers (from the amorphous state obtained by the CSM nanoindentation test). These values were compared with those for different BMG alloys reported in the literature. The full list of these alloys and the respective references is presented in Table S1 in the supplementary materials^71–86^.

The hardness and elastic modulus of the as-quenched and heat-treated DIN 1.4401 stainless steel microfibers were measured using continuous stiffness measurement (CSM) nanoindentation. The results are shown in Fig. 7. The reduced elastic modulus of the as-quenched amorphous DIN 1.4401 stainless steel microfibers was found to be within the range 103.5 ± 4.0 GPa, significantly lower than the elastic modulus of conventional DIN 1.4401 stainless steel (*E* = 193 ± 4 GPa). The elastic modulus gives a macroscopic measure of the stiffness of a material, and it reflects both interatomic bonding energies and atomic connectivity. Additionally, the hardness of the as-received DIN 1.4401 stainless steel increased from the original value of 3.5 ± 0.17 GPa to an average value of 7.8 ± 0.6 GPa for the as-cast (amorphous) stainless steel microfibers. This significant increase in hardness is related to the limited plastic deformation in metallic glasses in contrast to traditional crystalline alloys. Metallic glasses, due to their disordered nature, do not deform via dislocations that can accommodate plastic deformation at room temperature; instead, the deformations are inhomogeneous and depend on the formation of local clusters of atoms in what are commonly known as shear transformation zones (STZs), and they undergo inelastic shear distortions from one relatively low energy state to another low energy configuration^49,50^. The sheared entities finally evolve into localized shear bands carrying the deformation throughout the material^51^. Activation of STZs/shear bands requires higher energy than the initiation of dislocations in crystalline materials, which eventually increases the hardness^25^. The reduced elastic modulus of stainless steel (amorphous state) increased significantly to 199.8 ± 4.6 GPa after heat treatment (nanocrystalline state), and this was very close to the bulk elastic modulus of conventional DIN 1.4401 stainless steel. As discussed before, the elastic modulus is a macroscopic measure of stiffness and depends strongly on interatomic bonding. The same crystalline structure, regardless of grain size, means the atomic packing density is the same. Therefore, elastic modulus values close to those of traditional fcc austenitic stainless steel were expected. Moreover, the hardness of the stainless steel microfibers did not decrease after heat treatment but further increased to 13.5 ± 2.0 GPa. Improvements in hardness caused by nanocrystallization can be explained by the grain refinement phenomenon (Hall–Petch effect). Based on previous investigations of grain refinement and its effect on the mechanical properties of austenitic stainless steel, a hardness of 5.5 GPa was expected for stainless steel with an average grain size of 80 nm^52^, and the hardness measured for the nanocrystalline microstructure of the heat-treated stainless steel microfibers was more than twice that. Therefore, the local inhomogeneities observed for the microstructure (an amorphous layer covering nanocrystalline grains and an accumulation of certain elements in some grains observed by HRTEM), along with the Hall–Petch effect, could enhance the hardness. Kim et al.^53^ suggested that when nanocrystallites are too small to contain defects such as stacking faults or dislocations, the hardness and mechanical strength can increase as well.

As mentioned above, the presence of a very thin amorphous region close to the surface of the annealed DIN 1.4401 stainless steel microfibers could also have contributed to the high measured hardness. The thickness of this amorphous region was estimated to be approximately 100 nm. During nanoindentation (displacement depth of 250 nm) from the top surface of a microfiber, indentation and plastic deformation first went through this amorphous region. Eckert et al. suggested that formation of highly localized shear bands can be effectively suppressed by the interfaces formed by nanocrystallization^53^. Therefore, the presence of nanocrystals within the amorphous matrix, as well as adjacent grains, could be essential for increasing the hardness^54–56^. Overall, a combination of all the aforementioned mechanisms most likely contributes to this increased hardness.

Shear band confinement can change the deformation mode^9,57,58^ as long as the spacing of the second phase(s) matches the plastic zone size of the related BMG matrix^59–61^. This means that when the spacing of the second phase(s) (crystalline dendrites, voids or defects) is equal to or smaller than the plastic zone size (*R*_*P*_) of the material, shear bands will not immediately develop into cracks, but instead, formation of multiple shear bands is triggered, and thus, energy dispersion due to stress distribution is favored^62–65^. This can be described by^66^:

3 $${R}_{\mathrm{p}}=(1/2\pi ){({K}_{1C}/{\sigma }_{y})}^{2}$$

where ${R}_{\mathrm{p}}$ is the plastic zone size, ${K}_{1C}$ is the fracture toughness, and ${\sigma }_{y}$ is the yield strength of the considered metallic glass fiber. For a close composition based on Fe–Ni–Cr–Si–B (Fe_74_Ni_9_Cr_4_Si_3_B_10_, Fe_66_Ni_7_Zr_6_Cr_8_Si_3_B_10_, and Fe_63_Ni_7_Zr_6_Cr_8_W_3_Si_3_B_10_), ${\sigma }_{y}$ was measured to be 2930 MPa under compression ^67^. One can also assume that the minimum value of ${K}_{1C}$ for Fe-based metallic glasses is ~ 25 MPa m^1/2^^68^, which yields ${R}_{\mathrm{P}}\sim 11.5\mathrm{ \mu m}$. This value is much higher than the average microfiber thickness of 4 µm indicated by the SEM data. Hence, we believe that the relatively smaller sample thickness compared to the estimated intrinsic plastic zone size has an impact on the extensive hardening observed in the nanoindentation tests. The formation of shear bands accounts for the percolation of shear transformation zones (STZs). The STZ volume above which the initiation of plastic flow through cooperative shearing of unstable STZs is on the order of several nm^3^ (200–700 atoms)^69^. Therefore, the already formed shear bands will be stopped by the presence of very thin amorphous region close to the surface and thus leading to an increase in overall hardness.

Finally, we attempted to compare the elastic modulus and hardness measured for the conventional DIN 1.4401 stainless steel, the as-quenched stainless steel microfibers and the heat-treated stainless steel microfibers with values reported for different bulk metallic glass (BMG) systems, as presented in Fig. 7. The complete list of alloy compositions and their values is gathered in Table S1 in the supplementary materials. The hardness and elastic modulus of the as-cast amorphous stainless steel microfibers are quite close to the values measured for the Fe_80_P_13_C_7_ BMG, while the heat-treated stainless steel microfibers have values closer to the ((Fe_0.7_Co_0.3_)_0.75_B_0.2_Si_0.05_)_96_Nb_4_ BMG. Furthermore, the linear correlation between elastic modulus and hardness in BMGs is very clear in this figure. Macroscopic-scale plastic deformation in a metallic glass is essentially a biased accumulation of local strains triggered during the formation of STZs and redistribution of the free volume. If the flow (steady-state condition) is homogeneous, there is a balance between events creating and annihilating free volume. We have previously indicated that the elastic modulus in a metallic glass also depends on how densely it is packed. In other words, the availability of free volume determines the plastic response of the metallic glass. That is why the hardness and elastic modulus of these materials were almost linearly correlated. Furthermore, Chen et al.^70^ established a model showing that this linear correlation also holds for the correlation between bulk modulus and hardness for “intrinsically brittle materials”, which includes most BMGs since they fail in their elastic region.

## Discussion

In this work, we used a custom design based on the planar flow melt spinning device to fabricate stainless-steel microfibers. We have shown that the new technique makes it possible to fabricate metallic microfibers within a micrometer range in a single step process with a theoretical quenching rate of 10^8^ K.s^−1^. The as-quenched DIN 1.4401 stainless-steel microfibers showed a diffuse XRD peak, indicating the formation of an amorphous structure. This observation was further confirmed by HRTEM and FFT studies of the as-quenched microfibers. This is the first time that an amorphous structure has been generated from a commercial stainless-steel grade (DIN 1.4401). DSC measurements on the as-quenched microfibers showed a glass transition at 733 K and crystallization at 823 K, leading to a reduced glass transition temperature (T_rg_) of 0.43. This marginal number confirmed the rather poor glass-forming ability of stainless-steel and the need for very high cooling rates for glass formation. Subsequent heat treatment of the amorphous stainless-steel microfibers generated a composite structure comprising nanocrystalline grains with a thin amorphous layer on the top.

Continuous stiffness nanoindentation measurements conducted on as-quenched and annealed stainless-steel microfibers revealed a hardness increase from the original value of 3.5 ± 0.17 GPa for as-purchased bulk polycrystalline stainless-steel to 7.77 ± 0.6 GPa for melt-spun amorphous stainless-steel microfibers. Furthermore, the elastic modulus of the amorphous stainless-steel microfibers dropped from 199.8 ± 4.6 GPa for polycrystalline stainless-steel to approximately 103.5 ± 4.0 GPa. This reduction in elastic modulus suggested that the packing density in the generated amorphous phase was lower than the packing density of polycrystalline fcc (austenitic) stainless steel.

We further compared the elastic moduli and hardness values of previously reported metallic glasses with those of the present amorphous stainless-steel microfibers. There seems to be a linear correlation between the elastic modulus and hardness for most metallic glasses, as previously described by Chen et al.^70^.

## Methods

### Stainless steel microfiber fabrication

A DIN 1.4401 stainless steel alloy (Advent Research materials, England; X5CrNiMo17-12-2) was used to fabricate stainless steel microfibers with a custom-made melt spinning device. The melt-spinning device constituted a pure copper wheel, an induction coil, and a boron nitride (BN) crucible (18 × 95 mm) with a slit nozzle (10 × 0.03 mm^2^) (INNOVACERA, China) in a closed chamber. The chamber was evacuated to 10^–6^ mbar and further flushed with 800 mbar Ar (> 99.999%, purity). This step was repeated three times to prevent oxidation during the experiments. The linear speed of the copper wheel was fixed at 63 m.s^−1^, and its distance to the nozzle opening was fixed at 150 µm. After melting the stainless-steel rod at 1648 K, the melt was overheated up to 1823 K and ejected through the nozzle droplet-by-droplet into the 800 mbar Ar-filled chamber by applying a 1200 mbar Ar overpressure.

### Heat-treatment of stainless steel microfibers

The fabricated DIN 1.4401 microfibers were subsequently heat-treated in stepwise fashion at 20 K.min^−1^ to 773, 873, 973, and 1073 K; an isothermal holding time of 1 h was used for each step and was followed by furnace cooling in vacuum (Nabertherm GmbH, Germany).

### Characterization of stainless steel microfibers

Differential scanning calorimetry (DSC) tests were conducted using a Netzsch DSC 404 F1 Pegasus device (NETZSCH-Gerätebau GmbH, Germany) under a high purity (99.999%) Ar atmosphere at a constant heating and cooling rate of 20 K.min^−1^. The samples were heated twice in the DSC, and normalization was performed by subtracting the baseline from the original heating curve. The DSC tests were repeated three times and produced an error of ± 2 K for the glass transition and crystallization temperatures. X-ray diffraction measurements were conducted with a Panalytical Empyrean X-ray diffractometer with Co Kα radiation (*λ* = 17.089 nm; Co-Kα_1_ and Co-Kα_2_ radiation filtering) with a step size of 0.026° in theta−2 theta scan mode in the Bragg–Brentano geometry. The samples were directly used in the form of fiber bundles because powder formation from fibers could induce crystallization and phase transformations. Field emission scanning electron microscopy (SEM, JCM—6000Plus Versatile Benchtop JEOL, Tokyo, Japan) with an accelerating voltage between 10 and 15 kV was used for morphological analyses of stainless steel microfibers.

### TEM analysis

The microfibers were fabricated into transmission electron microscopy (TEM) cross-section samples using an FEI Helios 600 instrument with a standard lift out and polishing process and then ion milled in a Gatan 691 PIPS at 0.8 keV to remove residual contamination and possible damage. Bright field (BF) images, high-resolution electron microscopy (HREM) images, high-angle annular dark-field (HAADF) images and energy-dispersive X-ray spectroscopy (EDS) images were captured using a JEM Grand ARM300F microscope with double spherical aberration (Cs) correctors.

### Nanoindentation test

The microfibers were hot mounted by PolyFast at 453 K and 250 bar. They were ground using 1200, 2400 and 4000 SiC sandpapers and polished further with 1 μm diamond and alumina powder suspensions. Cleaning was performed using isopropanol and followed by air blow fast-drying. Nanoindentation tests were performed with an Agilent G200 Nanoindenter, and the results were analyzed using NanoVision software (Agilent Technologies, USA). The continuous stiffness method (CSM) was employed for the measurements. The following parameters were used for the measurements: Vickers indenter tip surface approach velocity: 10 nm.s^−1^, surface approach distance: 1000 nm, harmonic displacement: 2 nm with 45 Hz frequency, strain rate: 0.05 s^−1^, and a depth limit of 500 nm indentation was selected. Poisson’s ratio was considered to be 0.25 for the annealed (crystalline) stainless steel and 0.30 for the as-prepared amorphous stainless steel since Poisson’s ratios for Fe-based metallic glasses with similar compositions have been reported to be close to 0.30^87^.

## Supplementary Information

Supplementary Information.

## Data Availability

The datasets used and/or analysed during the current study available from the corresponding author on reasonable request.
