# Supplementary material for: Fabrication of stainless-steel microfibers with amorphous-nanosized microstructure with enhanced mechanical properties
Source: Sci Rep. 2022 Jun 24;12:10784. doi: 10.1038/s41598-022-14475-5 (PMC9232582; doi:10.1038/s41598-022-14475-5)
Supplement: Supplementary file 1 — Supplementary Information. [file 41598_2022_14475_MOESM1_ESM.pdf]

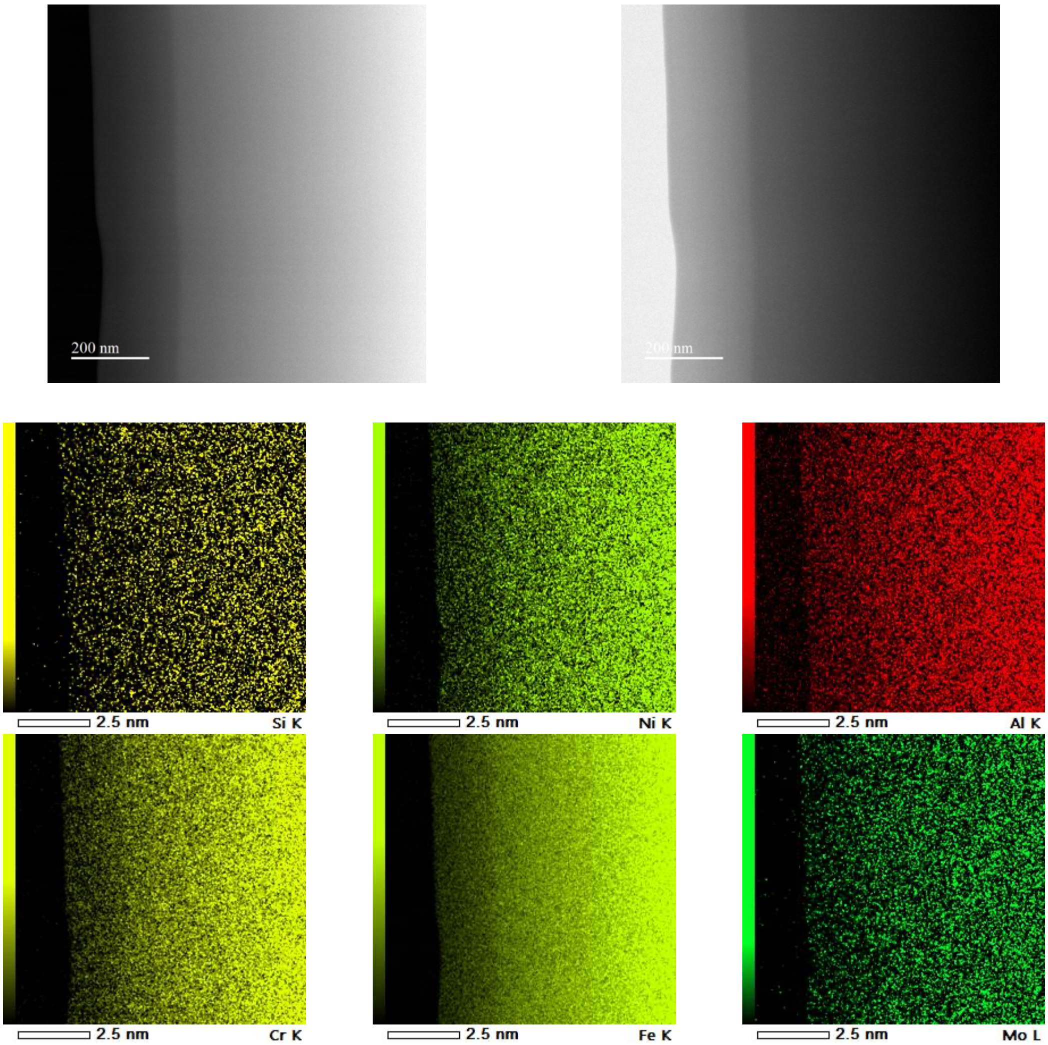

Figure S 1. Elemental map conducted on as-quenched DIN 1.4401 stainless steel microfibers cross-section.

Table S 1. List of MG compositions and their respected E and H which are used in Figure 7.

**Nr.**	**Alloy**	E (GPa)	H (GPa)	Ref
1	$Zr_{57}Ti_5Ni_8Cu_{20}Al_{10}$	85±1	4.6±0.1	(1)
2	$Hf_{11.4}Zr_{45.6}Ti_5Ni_8Cu_{20}Al_{10}$	87±1	4.7±0.1	(2)
3	$Hf_{22.8}Zr_{34.2}Ti_5Ni_8Cu_{20}Al_{10}$	94±1	5.2±0.1	(3)
4	$Zr_{52.5}Ti_5Ni_{14.6}Cu_{17.9}Al_{10}$	93±2	5.0±0.2	(3)
5	$Hf_{10.5}Zr_{42}Ti_5Ni_{14.6}Cu_{17.9}Al_{10}$	108±3	5.3±0.1	(3)

6	$Hf_{21}Zr_{31.5}Ti_5Ni_{14.6}Cu_{17.9}Al_{10}$	85±1	4.6±0.1	(3)
7	$Hf_{31.5}Zr_{21}Ti_5Ni_{14.6}Cu_{17.9}Al_{10}$	110±3	5.8±0.2	(3)
8	$Hf_{42}Zr_{10.5}Ti_5Ni_{14.6}Cu_{17.9}Al_{10}$	124±2	6.0±0.2	(3)
9	$Hf_{52.5}Ti_5Ni_{14.6}Cu_{17.9}Al_{10}$	128±4	6.1±0.2	(3)
10	$Zr_{57}Nb_5Ni_{12.6}Cu_{15.4}Al_{10}$	89±1	4.9±0.1	(3)
11	$(Al_{0.84}Y_{0.09}Ni_{0.05}Co_{0.02})_{95}Sc_5$	78	4.4	(4)
12	$(Al_{0.85}Y_{0.08}Ni_{0.05}Co_{0.02})_{95}Sc_5$	75	4.6±4.00.1	(4)
13	$La_{55}Al_{25}Cu_{10}Ni_5Co_5$	41.9	3	(5)
14	$Pr_{55}Al_{25}Co_{20}$	45.9	2.58	(5)
15	$Fe_{80}\ B_{20}$	167	10.6	(6)
16	$Fe_{80}P_{13}C_7$	118	7.5	(7)
17	$Fe_{78}\ B_{10}\ Si_{12}$	118	8.9	(7)
18	$(Fe_{0.75}B_{0.2}Si_{0.05})_{96}\ Nb_4$	180	10.5	(8)
19	$((Fe_{0.9}Co_{0.1})_{0.75}B_{0.2}Si_{0.05})_{96}Nb_4$	190	11.3	(8)
20	$((Fe_{0.8}Co_{0.2})_{0.75}B_{0.2}Si_{0.05})_{96}Nb_4$	205	12.0	(8)
21	$((Fe_{0.7}Co_{0.3})_{0.75}B_{0.2}Si_{0.05})_{96}Nb_4$	210	12.2	(8)
22	$((Fe_{0.6}Co_{0.4})_{0.75}B_{0.2}Si_{0.05})_{96}Nb_4$	210	12.3	(8)
23	$((Fe_{0.5}Co_{0.5})_{0.75}B_{0.2}Si_{0.05})_{96}Nb_4$	210	12.0	(8)
24	$Ni_{45}Ti_{20}Zr_{25}Al_{10}$	114	7.8	(9)
25	$Ni_{40}Cu_6Ti_{16}Zr_{28}Al_{10}$	111	7.6	(9)
26	$Ni_{40}Cu_5Ti_{17}Zr_{28}Al_{10}$	134	8.4	(9)
27	$Ni_{40}Cu_5Ti_{16.5}Zr_{28.5}Al_{10}$	122	7.8	(9)
28	$Ni_{39.8}Cu_{5.97}Ti_{15.92}Zr_{27.86}Al_{9.95}Si_{0.5}$	11	8.1	(9)
29	$Zr_{41.25}Ti_{13.75}Ni_{10}Cu_{12.5}Be_{22.5}$	96	5.2	(10)
30	$Pd_{80}Si_{20}$	67	3.2	(10)

31	$Pd_{40}Ni_{40}P_{20}$	108	5.3	(11)
32	$Pd_{77.5}Cu_{6}Si_{16.5}$	88	4.9	(11)
33	$Fe_{41}Co_{7}Cr_{15}Mo_{14}C_{15}B_{6}Y_{2}$	226	12.57	(12)
34	$Ni_{50}Nb_{50}$	132	8.93	(13)
35	$[Zr_{41}Ti_{14}Cu_{12.5}Ni_{10}Be_{22.5}]_{98}Y_{2}$	107.6	6.76	(14)
36	$Zr_{54}Al_{15}Ni_{10}Cu_{19}Y_{2}$	92.1	6.49	(14)
37	$Zr_{53}Al_{14}Ni_{10}Cu_{19}Y_{4}$	86	6.44	(14)
38	$Zr_{41}Ti_{14}Cu_{12.5}Ni_{8}Be_{22.5}C$	106	6.13	(14)
39	$Zr_{46.75}Ti_{8.25}Cu_{7.5}Ni_{10}Be_{27.5}$	100	6.1	(13)
40	$Zr_{48}Nb_{8}Cu_{14}Ni_{12}Be_{18}$	93.7	6.09	(14)
41	$Zr_{34}Ti_{15}Cu_{10}Ni_{11}Be_{28}Y_{2}$	109.8	6.07	(14)
42	$Zr_{57}Nb_{5}Cu_{15.4}Ni_{12.6}Al_{10}$	87.3	5.9	(13)
43	$Zr_{48}Nb_{8}Cu_{12}Fe_{8}Be_{24}$	95.7	5.85	(14)
44	$Zr_{40}Ti_{15}Cu_{11}Ni_{11}Be_{21.5}Y_{1}Mg_{0.5}$	94.2	5.74	(14)
45	$Zr_{41}Ti_{14}Cu_{12.5}Ni_{10}Be_{22.5}$	101	5.97	(13),(14)
46	$Zr_{41}Ti_{14}Cu_{12.5}Ni_{10}Be_{22.5}$	101	5.4	(13), (15)
47	$Zr_{41}Ti_{14}Cu_{12.5}Ni_{10}Be_{22.5}$	101	5.88	(13), (16)
48	$Zr_{41}Ti_{14}Cu_{12.5}Ni_{10}Be_{22.5}$	101	5.23	(13)
49	$Zr_{65}Al_{10}Ni_{10}Cu_{15}$	83	5.6	(13)
50	$Zr_{65}Al_{10}Ni_{10}Cu_{15}$	83	5.6	(13)
51	$Zr_{57}Ti_{5}Cu_{20}Ni_{8}Al_{10}$	82	5.4	(13)
52	$Cu_{60}Hf_{10}Zr_{20}Ti_{10}$	101	7	(13)
53	$Cu_{50}Zr_{50}$	88.7	5.8	(13)
54	$Cu_{50}Zr_{50}$	85	5.8	(13)
55	$Cu_{50}Zr_{45}Al_{5}$	102	5.4	(13)
56	$Pd_{40}Ni_{40}P_{20}$	108	5.38	(13)
57	$Pd_{40}Ni_{40}P_{20}$	108	5.3	(13)

58	$Pd_{40}Ni_{10}Cu_{30}P_{20}$	98	5	(13)
59	$Pd_{77.5}Si_{16.5}Cu_{6}$	92.9	4.5	(13)
60	$Pd_{77.5}Si_{16.5}Cu_{6}$	96	4.5	(13)
61	$Pt_{60}Ni_{15}P_{25}$	96	4.1	(13)
62	$Mg_{65}Cu_{25}Tb_{10}$	51.3	2.83	(13)
63	$Nb_{60}Al_{10}Fe_{20}Co_{10}$	51.2	2.2	(13)
64	$Ce_{70}Al_{10}Ni_{10}Cu_{10}$	30	1.5	(13)
65	$Er_{55}Al_{25}Co_{20}$	70.72	5.45	(5)
66	$Dy_{55}Al_{25}Co_{20}$	61.36	4.7	(5)
67	$Tb_{55}Al_{25}Co_{20}$	59.53	4.42	(5)
68	$Ho_{55}Al_{25}Co_{20}$	66.64	4.14	(5)

1. Zhang H, Subhash G, Kecskes LJ, Dowding RJ. Mechanical behavior of bulk (ZrHf)TiCuNiAl amorphous alloys. Scr Mater [Internet]. 2003 Sep;49(5):447–52. Available from: https://linkinghub.elsevier.com/retrieve/pii/S1359646203002951
2. Li H, Subhash G, Gao X-L, Kecskes LJ, Dowding RJ. Negative strain rate sensitivity and compositional dependence of fracture strength in Zr/Hf based bulk metallic glasses. Scr Mater [Internet]. 2003 Dec;49(11):1087–92. Available from: https://linkinghub.elsevier.com/retrieve/pii/S1359646203005189
3. Zhang HW, Subhash G, Jing XN, Kecskes LJ, Dowding RJ. Evaluation of hardness-yield strength relationships for bulk metallic glasses. Philos Mag Lett. 2006;
4. Inoue A, Sobu S, Louzguine D V, Kimura H, Sasamori K. Ultrahigh strength Al-based amorphous alloys containing Sc. J Mater Res. 2004;
5. Li S, Wang RJ, Pan MX, Zhao DQ, Wang WH. Formation and properties of RE55Al25Co20 (RE = Y, Ce, La, Pr, Nd, Gd, Tb, Dy, Ho and Er) bulk metallic glasses. J Non Cryst Solids. 2008;
6. Makino A, Kubota T, Makabe M, Chang CT, Inoue A. FeSiBP metallic glasses with high glass-forming ability and excellent magnetic properties. Mater Sci Eng B Solid-State Mater Adv Technol. 2008;
7. Davies HAA, Luborsky FE, Liebermann HH, Finney JLL, Wagner CNJ, Suzuki K, et al. Subject index. Vol. 33, Amorphous Metallic Alloys. Butterworth & Co (Publishers) Ltd.; 1983. 1–7 p.
8. Inoue A, Shen BL, Chang CT. Super-high strength of over 4000 MPa for Fe-based bulk glassy alloys in [(Fe1-xCox)0.75B0.2Si 0.05]96Nb4 system. Acta Mater. 2004;
9. Xu D, Duan G, Johnson WL, Garland C. Formation and properties of new Ni-based amorphous alloys with critical casting thickness up to 5 mm. Acta Mater [Internet]. 2004 Jul;52(12):3493–7. Available from: https://linkinghub.elsevier.com/retrieve/pii/S1359645404002058
10. Conner RD, Dandliker RB, Johnson WL. Mechanical properties of tungsten and steel fiber reinforced Zr41.25Ti13.75Cu12.5Ni10Be22.5 metallic glass matrix composites. Acta Mater [Internet]. 1998 Nov;46(17):6089–102. Available from:

https://linkinghub.elsevier.com/retrieve/pii/S1359645498002754
11. Donovan PE, Stobbs WM. The structure of shear bands in metallic glasses. Acta Metall [Internet]. 1981 Aug;29(8):1419–36. Available from: https://linkinghub.elsevier.com/retrieve/pii/0001616081901772
12. Keryvin V, Hoang VH, Shen J. Hardness, toughness, brittleness and cracking systems in an iron-based bulk metallic glass by indentation. Intermetallics [Internet]. 2009 Apr;17(4):211–7. Available from: https://linkinghub.elsevier.com/retrieve/pii/S0966979508002501
13. Wang WH. Correlations between elastic moduli and properties in bulk metallic glasses. J Appl Phys [Internet]. 2006 May;99(9):093506. Available from: http://aip.scitation.org/doi/10.1063/1.2193060
14. Zhang Y, Zhao DQ, Pan MX, Wang WH. Glass forming properties of Zr-based bulk metallic alloys. J Non Cryst Solids. 2003;315(1–2):206–10.
15. Wang WH, Bai HY. Carbon-addition-induced bulk ZrTiCuNiBe amorphous matrix composite containing ZrC particles. Mater Lett [Internet]. 2000 May;44(1):59–63. Available from: https://linkinghub.elsevier.com/retrieve/pii/S0167577X99002840
16. Narayan RL, Boopathy K, Sen I, Hofmann DC, Ramamurty U. On the hardness and elastic modulus of bulk metallic glass matrix composites. Scr Mater [Internet]. 2010 Oct;63(7):768–71. Available from: https://linkinghub.elsevier.com/retrieve/pii/S1359646210003957
